# Methylethynyl-Terminated Polyimide Nanofibrous Membranes: High-Temperature-Resistant Adhesives with Low-Temperature Processability

**DOI:** 10.3390/polym14194078

**Published:** 2022-09-28

**Authors:** Haoran Qi, Xi Ren, Yuang Liu, Shengwei Dai, Changxu Yang, Xiaolei Wang, Jingang Liu

**Affiliations:** School of Materials Science and Technology, China University of Geosciences, Beijing 100083, China

**Keywords:** polyimide, electrospinning, methylethynyl, adhesives, lap shear strength

## Abstract

As an alternative to traditional riveting and welding materials, high-temperature-resistant adhesives, with their unique advantages, have been widely used in aviation, aerospace, and other fields. Among them, polyimide (PI) adhesives have been one of the most studied species both from basic and practical application aspects. However, in the main applications of solvent-type PI adhesives, pinholes or bubbles often exist in the cured PI adhesive layers due to the solvent volatilization and dehydration reaction, which directly affect the adhesive performance. To address this issue, electrospun PI nanofibrous membranes (NFMs) were employed as solvent-free or solvent-less adhesives for stainless steel in the current work. To enhance the adhesion of PI adhesives to the metal substrates, phenolphthalein groups and flexible ether bonds were introduced into the main chain of PIs via the monomers of 4,4′-oxydiphthalic anhydride (ODPA) and 3,3-bis[4-(4-aminophenoxy)phenyl] phthalide (BAPPT). At the same time, the methylethynyl group was used as the end-capping component, and the crosslinking reaction of the alkynyl group at high temperature further increased the adhesive strength of the PI adhesives. Three kinds of methylethynyl-terminated PI (METI) NFMs with the set molecular weights of 5000, 10,000, and 20,000 g/mol were first prepared via the one-step high-temperature polycondensation procedure. Then, the PI NFMs were fabricated via the standard electrospinning procedure from the soluble METI solutions. The afforded METI NFMs showed excellent melt-flowing behaviors at high temperature. Incorporation of the methylethynyl end-capping achieved a crosslinking reaction at 280−310 °C for the NFMs, which was about 70 °C lower than those of the phenylacetylene end-capping counterparts. Using the METI NFMs as adhesive, stainless steel adherends were successfully bonded, and the single-lap shear strength (LSS) was higher than 20.0 MPa at both room temperature (25 °C) and high temperature (200 °C).

## 1. Introduction

High-temperature-resistant adhesives generally refer to a class of special adhesives that can be used for a long time at hundreds of degrees Celsius or can withstand thousands of degrees Celsius in a short time [1]. Due to the better fatigue resistance and higher strength-to-weight ratio, high-temperature polymer adhesives have been widely used in the aerospace field to replace traditional connection methods such as riveting, welding, and mechanical fastening [2,3]. Among various high-temperature polymers, heteroaromatic polymers can usually endow adhesives with excellent thermal stability due to the highly conjugated structural characteristics and strong intermolecular interactions [4]. Polyimide (PI) adhesives represent a class of the most widely studied heteroaromatic polymer adhesives due to high bonding strength, good processing performance, and good mechanical properties [5,6,7,8]. At present, the main problems of the currently used PI adhesives are mainly focused on the high porosity in the cured adhesion layers. For example, either the soluble PI (SPI) types [9,10] or the poly(amic acid) (PAA) types [11] of PI adhesives usually produce pinholes or voids in the cured adhesion layers due to solvent evaporation and dehydration reactions during high-temperature imidization. This inevitably reduces the bonding strength of the adherends. As for film-type PI adhesives [12,13] and powder-type PI adhesives [14,15] with fewer contents of solvents, the former is usually limited to planar bonding while the latter is more difficult to apply. In addition, for conventional thermosetting PI adhesives with rigid molecular chains and reactive end-capping groups, there are usually problems with high curing temperatures, which increase the processing difficulty and limit the applications in temperature-sensitive fields. For example, the 5-norbornene-2,3-dicarboxylic acid anhydride (NA)-end-capped LARC-13 [16] developed by the National Aeronautics and Space Administration (NASA), USA, has a curing temperature of 343 °C; the ethynyl-terminated Thermid 600 [17], developed by the American Hughes Aircraft Company, has a curing temperature of 340−350 °C; the phenylethynyl-terminated PETI-5 [18], developed by NASA, USA, has a curing temperature over 370 °C. Therefore, the question of how to realize the solvent-free adhesion of PI adhesives at reduced curing temperature has become one of the most attractive topics in the research field of high-performance PI adhesives [19].

PI nanofibrous membranes (NFMs) prepared by an electrospinning procedure have been widely used in various fields due to their excellent combined properties [20]. Based on our previous research on the research and development of high-performance PI NFMs [21,22,23,24,25], it can be found that the solvent-free characteristics, good flexibility, highly entangled microstructure, and good processability of the PI NFMs make them good candidates for high-performance adhesives. Thus, it could be anticipated that electrospun PI NFMs might find wide applications as high-temperature-resistant adhesives. In our previous work, a series of phenylethynyl-terminated PI (PETI) NFMs were prepared and characterized as adhesives [26]. Although the developed PI NFM adhesives exhibited good adhesion properties, adhesion temperatures over 370 °C limit their wide application in practice.

In the current work, a series of methylethynyl-terminated PI (METI) NFMs were designed and developed as high-temperature adhesives in order to reduce the crosslinking temperatures of PETI analogues. The phenolphthalein groups were maintained in the molecular chains of the developed METI NFMs due to the large molar volume, asymmetric molecular structure, and potential crosslinking features [27,28]. The structure-property relationships of the developed METI NFM adhesives were investigated in detail.

## 2. Materials and Methods

### 2.1. Materials

4,4′-Oxydiphthalic anhydride (ODPA, Changzhou Sunshine Pharmaceutical Co., Ltd., Changzhou, China) and 4-methylethynyl phthalic anhydride (MEPA, Nexam Chemical, Lomma, Sweden) were dried at 180 °C and 120 °C for 10 h, respectively, prior to use. 3,3-bis[4-(4-aminophenoxy)phenyl]phthalide (BAPPT) was synthesized in our laboratory according to the literature [29]. Electronic-grade *N,N*-dimethylacetamide (DMAc), *N,N*-dimethylformamide (DMF), and *N*-methylpyrrolidone (NMP), purity ≥ 99.8%, Na^+^ ≤ 2 ppm, water content ≤ 200 ppm (Grinda Electronic Materials Co., Ltd., Hangzhou, China) were used directly.

### 2.2. Characterization and Methods

The inherent viscosity of METI resins was measured at 25 °C using an Ubbelohde viscometer (As-one, Osaka, Japan), and the absolute viscosity was measured at 25 °C using a DV2-TRVCP viscometer (Brookfield, Middleboro, MA, USA). Molecular weights of METI resins, including number-average molecular weight (M_n_) and weight-average molecular weight (M_w_), were determined using an LC-20AD-type gel permeation chromatography (GPC, Shimadzu Corporation, Kyoto, Japan) system. Solubility tests were characterized as follows: 1.0 g of METI resins was mixed with 9.0 g of solvent (solid content 10 wt%) and stirred at room temperature for 24 h. Its solubility was visually judged and divided into three grades: completely dissolved (++), partially dissolved (+−), and insoluble (−), where complete dissolution means that the resin is uniformly dispersed in the solution, transparent, without phase separation, precipitation, or gel formation; insolubilization indicates no change in the appearance of the resin.

Attenuated total reflection–Fourier transform infrared (ATR-FTIR) spectra of PI NFMs were measured by a Bruker Tensor-27 FT-IR spectrometer (Bruker, Billerica, MA, USA), wavenumber range: 4000−400 cm^−1^. Wide-angle X-ray diffraction (XRD) spectra were measured with a D/max-2500 X-ray diffractometer (Rigaku Corporation, Tokyo, Japan), Cu-Kα1 radiation, operating at 40 kV and 200 mA. Field-emission scanning electron microscope (FE-SEM) imaging was performed using a JSM-6700F (JEOL, Tokyo, Japan) with an accelerating voltage of 15 kV, and Pt/Pd was sputtered on each film before measurement. Ultraviolet–visible spectroscopy (UV–Vis) was performed at room temperature using a U-3210 spectrophotometer (Hitachi, Tokyo, Japan) for testing, and the PI fibrous membrane was dried at 100 °C for 10 h before the test to remove the adsorbed moisture.

Thermogravimetric analysis (TGA) of PI NFMs was tested by a TA-Q50 thermal analysis system (Perkin-Elmer, Waltham, MA, USA) at a test temperature range of 30–760 °C and a heating rate of 20 °C/min; the test environment was nitrogen and the gas flow rate was 20 mL/min. Differential scanning calorimetry (DSC) was tested with a DSC 214 calorimetric differential scanner (NETZSCH, Selb, Germany); the test temperature range was 30−400 °C, the heating rate was 10 °C/min, and the test environment was nitrogen with a gas flow of 20 mL/min. The rheological evaluation was carried out on an AR2000 rheometer (TA Instrument, Newcastle, DE, USA). METI powder was pressed to obtain a sample disk (diameter: 25 mm, thickness: 1.5 ± 0.2 mm). During the measurement, METI discs were loaded into parallel plates. The top parallel plate oscillated at a fixed angular frequency of 0.5 Hz and a fixed strain of 1.0%; the data acquisition range was 100−440 °C and the heating rate was 4 °C/min. The single-lap shear strength (LSS) test was performed on an Instron 5567 tensile machine (Instron, Boston, MA, USA). The elongation speed was 2 mm/min.

### 2.3. Synthesis of METI Resins

Synthesis of soluble PI resin: METI resin was prepared by a high-temperature polycondensation method, using ODPA, BAPPT, and MEPA as raw materials, by controlling the ratio of the three raw materials. METI resins with molecular weights of 5000 g/mol, 10,000 g/mol, and 20,000 g/mol were designed and named METI-5K, 10K, and 20K, respectively. The following presentation takes METI-20K as an example to introduce the synthesis process of METI resin in detail: BAPPT (19.3218 g, 38.6019 mmol) and NMP (100 g) were charged into a 500 mL three-necked flask equipped with a mechanical stirrer, an electric heating bath, and a Dean–Stark trap and purged with nitrogen at room temperature, then ODPA (11.5094 g, 37.1019 mmol) was added to the solution and stirred for 5 h. MEPA (0.5585 g, 3 mmol) and NMP (25 g) were added at the same time, and the solid content of the reaction mixture was controlled to be about 20 wt%. After stirring for another 14 h at room temperature, toluene (100 g) and isoquinoline (1.0 g) were then added to the solution after stirring for a further 14 h at room temperature. The reaction mixture was heated to 140−145 °C, and the by-product water generated by the reaction was removed by toluene/water azeotrope. The refluxing dehydration reaction was maintained for 16 h. Then, toluene was distilled from the reaction system until the internal temperature of the reaction reached 180 °C, and the reaction was carried out at 180 °C for 1 h. The solution was cooled to 70 °C and the resulting viscous solution was precipitated in excess aqueous ethanol (10 wt%). Finally, the precipitated METI-20K resin was dried at room temperature for 24 h, and then dried under vacuum at 130 °C for 24 h. Finally, white filamentous METI-20K resin was obtained, yield: 29.62 g (97.7%).

The METI-10K and METI-5K resins were prepared according to a procedure similar to the one mentioned above, with the formula shown in Table 1. METI-5K: yield: 28.76 g (95.8%). METI-10K: yield: 29.08 g (96.9%).

In addition, PI-ref (ODPA-BAPPT) resin without end-capping was synthesized according to the above method, and the reactant amounts of BAPPT and ODPA were 26.0781 g (52.1 mmol) and 16.1725 g (52.1 mmol), respectively. Yield: 39.41 g (97.3%).

### 2.4. Preparation of METI NFMs

Taking METI-20K NFM as an example, the detailed preparation process of METI NFMs is as follows: The completely dried METI-20K resin was dissolved in DMAc solution, and the solid content of the solution was controlled at 33 wt% to obtain a solution with an absolute viscosity of 5000 mPa s. The obtained METI-20K solution was uniformly injected into a 5 mL syringe using a spinneret with an inner diameter of 0.21 mm. The METI-20K solution was extruded from the spinneret with a syringe pump at a rate of 0.2 mL/h. The positive voltage between the injector and the collector was 16 kV and the negative voltage was −3 kV. The distance between the spinneret and the grounded drum collector (diameter: 10 cm; length: 30 cm) was 15 cm. The relative humidity of the electrospinning device was controlled at 30 ± 5%, and the speed of the collector was set at 2 rpm. METI-20K fibers were densely wound and deposited on aluminum foil. The resulting METI-20K NFM was vacuum-dried at 120 °C for 1 h to remove residual solvent.

METI-5K and METI-10K NFMs were prepared following the same procedure as above, but starting with either a METI-5K or METI-10K solution with a solid content of 47.5 wt% or 38 wt% (absolute viscosity: ~5000 mPa s), respectively, in the electrospinning solution.

### 2.5. Preparation of Stainless Steel Samples Adhered with the METI NFMs

The stainless steel adherends (100 mm × 25.4 mm × 2 mm, length × width × height) used for the lap shear stress (LSS) test were pretreated by polishing with standard sandpaper before use. The METI UFMs were cut into small pieces (12.5 mm × 25.4 mm, length × width), stacked, and placed between two stainless steel adherends. The thickness of the final cured METI NFMs was controlled to be 0.3−0.5 mm. The overlapping area was 12.5 mm × 25.4 mm (length × width). The METI NFMs were tightly clamped to the overlap of two stainless steel sheets. Subsequently, the METI NFM-adhered specimens were placed into an oven and then heated at 80 °C, 150 °C, 220 °C, 290 °C, 310 °C, and 330 °C each for 1 h. Then, the samples were cooled to room temperature. The LSS values at room temperature (25 °C) were measured according to standard of GB/T 7124-2008 [30], “Determination of tensile shear strength of adhesives (rigid material vs. rigid material)”. The high-temperature (200 °C) LSS tests were carried out according to the standard of GJB 444-1988 [31], “Test method for high temperature tensile shear strength of adhesives (metal to metal)”. In each LSS test, five specimens were tested and the average data were recorded.

## 3. Results and Discussion

### 3.1. Molecular Weight and Solubility

**Figure 1:**
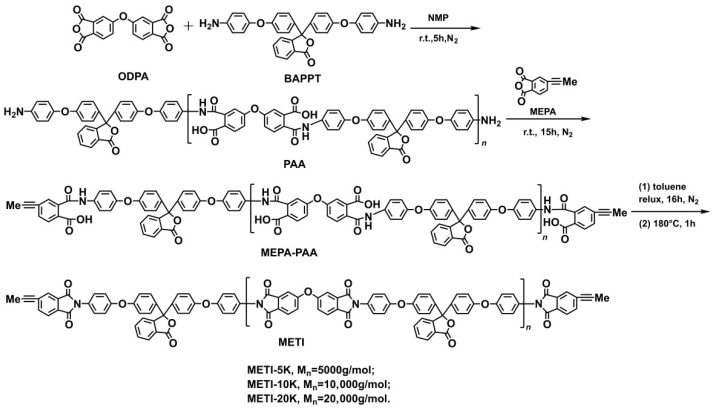
Preparation of METI resins.

The preparation process of three METI resins is shown in Figure 1. The METI resins were named “METI-5K”, “METI-10K”, and “METI-20K” for the systems with designed molecular weights of 5000 g/mol, 10,000 g/mol, and 20,000 g/mol, respectively. First, the methylethynyl-terminated polyamic acid (MEPA-PAA) was synthesized using ODPA, BAPPT, and MEPA as the starting materials. Then, the soluble METI resins were afforded after dehydration at 180 °C. At last, METI-20K was obtained as continuous fibrous resin, METI-10K was obtained as short rod-like resin, and METI-5K was obtained as granular resin, indicating the gradually decreasing molecular weights of the resins.

The M_n_ values of the METI resins shown in Table 2 are 1.59 × 10^4^ g/mol for METI-5K, 1.71 × 10^4^ g/mol for METI-10K, and 3.53 × 10^4^ g/mol for METI-20K. It can be deduced from the data that all the measured M_n_ values were higher than the designed values. This is consistent with the literature [32,33], which is mainly because the lithium bromide (LiBr) and phosphoric acid (H_3_PO_4_) in the GPC mobile phase affect the solubility of METI resins in NMP, resulting in an increase in the measured values. The METI resins exhibited narrow PDI values of 1.36 for METI-5K, 2.12 for METI-10K, and 1.66 for METI-20K. In addition, the [η]_inh_ and the M_n_ values of the controlled PI-ref (ODPA-BAPPT) resin were 1.21 dL/g and 19.0 × 10^4^ g/mol, respectively, indicating the high reactivity of the monomers. The moderate-to-high molecular weight feature of the METI resins, on one hand, could afford good melting flowability at elevated temperatures and, on the other hand, could guarantee good mechanical properties of the afforded electrospun NFMs.

All three METI resins exhibited good solubility in polar aprotic solvents, as shown in Table 1. They were all soluble in NMP, DMAc, and DMF at room temperature with a solid content of 10%. They were also partially soluble in chloroform (CHCl_3_) and tetrahydrofuran (THF). The PI-ref resin was insoluble in tetrahydrofuran. The good solubility of the METI resins in the test solvents, on one hand, came from the controlled molecular weights and, on the other hand, from the flexible ether linkages and bulky phenolphthalein groups in the molecular chains of the resins.

**Figure 2:**
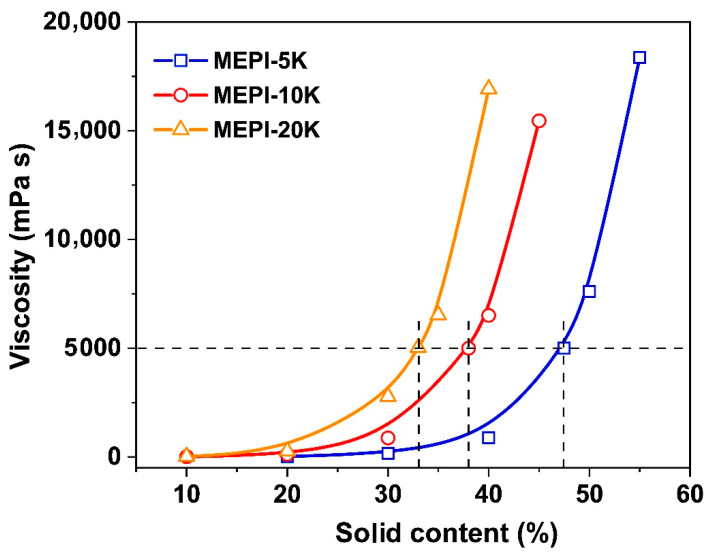
Viscosity-solid content relationship of METI solutions with DMAc as solvent at room temperature.

The viscosity-solid content relationship curves of the three METI resins are shown in Figure 2. There are clear differences in the solubility of the METI resins with different molecular weights in the same DMAc solvent. It can be seen that the viscosity of the METI resins gradually decreased with the decreasing molecular weights at the same solid content. In order to afford PI NFMs with fine micromorphologies, the METI solutions with the absolute viscosities of 5000 mPa s were prepared for the following electrospinning fabrications. According to the figure, different solid contents that were 47.5 wt% for METI-5 K, 38.0 wt% for METI-10 K, and 33.0 wt% for METI-20 K were chosen.

### 3.2. Electrospinning Fabrications of METI NFMs and the Micromorphology

**Figure 3:**
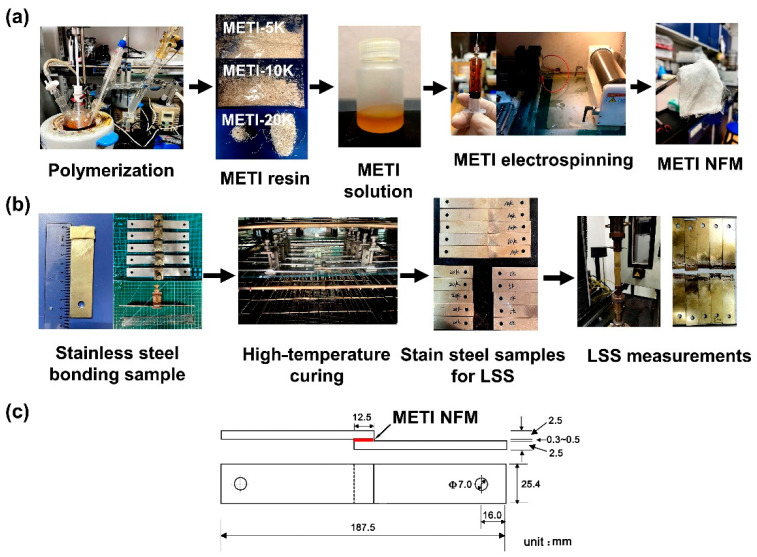
(**a**) Electrospinning process for METI NFMs; (**b**) Fabrication of stainless steel adherends and the lap shear strength (LSS) measurements; (**c**) Schematic diagram of the dimensions of stainless steel adherends for adhesion.

**Figure 4:**
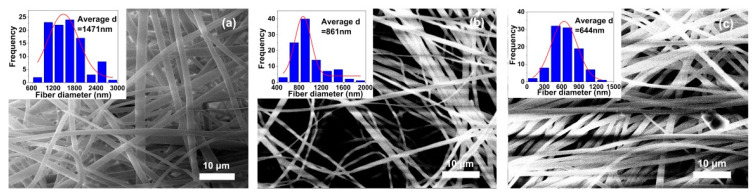
SEM images and average diameters (d_av_) of METI NFMs. (**a**) METI-5K; (**b**) METI-10K; (**c**) METI-20K.

A series of METI NFMs were fabricated according to the electrospinning procedures shown in Figure 3 with the parameters shown in Section 2.4. It can be seen from the SEM images of the METI NFMs, shown in Figure 4, that the NFMs with fine micromorphology were successfully prepared. The flexible and tough fibrous membranes were composed of tangled nanofibers with average diameters (d_av_) from 644 nm (METI-20K) to 1471 nm (METI-5K). At the same viscosity, with a decrease in the designed molecular weights, the d_av_ values gradually increased. This might be attributed to the higher solid content of the resin solution with lower molecular weights at the same viscosity, resulting in higher resin content in the spinning solution sprayed from the spinneret per unit of time. Macroscopically, the d_av_ values became larger.

**Figure 5:**
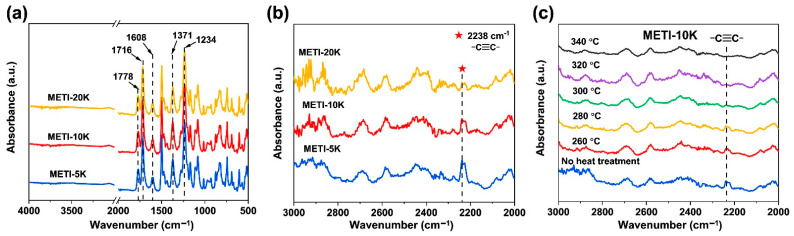
FTIR spectra of METI NFMs. (**a**) spectra of pristine METI NFMs; (**b**) spectra in the wavenumber range of 2000−3000 cm^−1^; (**c**) spectra of METI-10K treated at different temperatures.

To confirm the chemical structures of the METI NFMs, FTIR measurements were performed on the fibrous membranes with a reflective mode. Figure 5a reveals the FTIR spectra of the pristine NFMs without heat treatment. The carbonyl asymmetric stretching vibration peak at 1778 cm^−1^, the asymmetric stretching vibration peak at 1716 cm^−1^, and the C–N bond stretching vibration peak at 1371 cm^−1^ clearly prove the existence of the imide rings. Secondly, the phenolphthalein –C(O)–O– asymmetric stretching vibration peak at 1608 cm^−1^ and the characteristic absorption peaks of the ether bonds (–O–) at 1234 cm^−1^ were also detected. Since the contents of alkynyl groups (C≡C) in METIs were quite low and the absorption peaks were weaker, the absorption of the C≡C groups in the wavenumber range of 2000−3000 cm^−1^ was enlarged, shown in Figure 5b. Obvious stretching vibration peaks of the substituted –C≡C– at 2238 cm^−1^ were detected for all METI NFMs. Visually, the intensity of the –C≡C– absorption peaks gradually decreases with increasing molecular weights for the polymers, which is mainly related to the decrease in alkynyl contents in the polymers. The above infrared absorption peaks demonstrate the successful preparation of METI NFMs.

Furthermore, the METI-10K NFM was heat-treated at different temperatures, and the FTIR spectra are shown in Figure 5c. With increasing temperatures, the characteristic absorption peaks of –C≡C– groups gradually weakened and nearly disappeared at about 300 °C, which was mainly due to the crosslinking of the alkynyl groups at this temperature [34]. Thus, it can be roughly determined that the crosslinking reaction for the current METI-10 NFM occurred at around 300 °C. Obtained by the same method, the crosslinking temperatures of METI-20K and METI-5K samples were about 320 °C and 280 °C, respectively.

### 3.3. Thermal Properties

**Figure 6:**
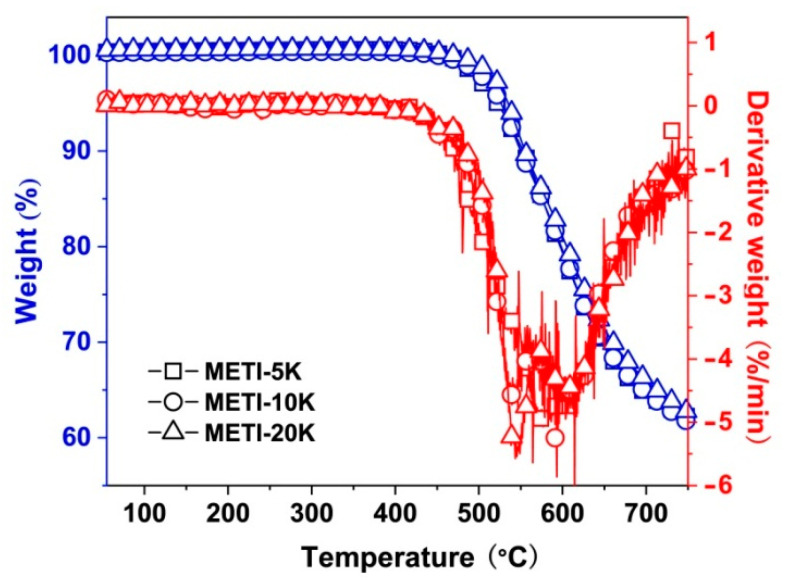
TGA and DTG plots of METI NFMs in nitrogen atmosphere.

Two methods, TGA and DSC, were used to investigate the thermal stability of the METI NFMs at elevated temperatures and the data are summarized in Table 3. Figure 6 records the TGA and derivative TGA (DTG) curves of the METI NFMs. All three METI NFMs exhibit good thermal stability up to 450 °C. With a further increase in temperature, the NFMs began to decompose, reaching a 5% weight-loss temperature (T_5%_) of 522.5 °C for METI-5K, 520.2 °C for METI-10K, and 534.8 °C for METI-20K. From the DTG curve, it can be clearly seen that METI-10K and METI-20K samples exhibited clear two-stage thermal decomposition behaviors. The first stage occurred at about 540 °C, which was mainly due to the decomposition of the side-chain phthalide groups. The second stage was recorded at about 600 °C, which was mainly attributed to decomposition of the main chains of the polymers. Two-stage decomposition was not observed for METI-5K, which might be due to the reduced thermal resistance caused by lower molecular weights [35]. The 700 °C residual weight ratio (R_w700_) of the METI NFMs was about 64.6–65.9 wt%. As expected, METI-20K NFM, with the highest molecular weights, showed the highest R_w700_ value.

**Figure 7:**
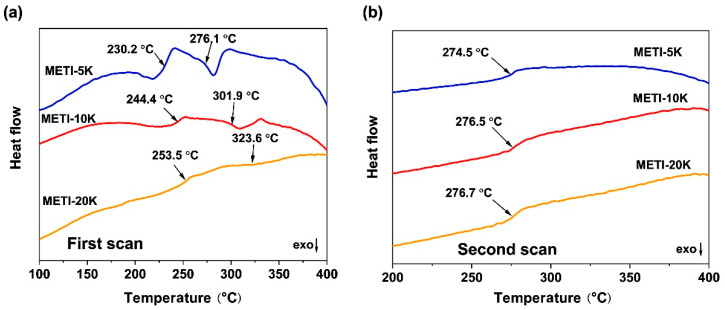
DSC diagrams of METI NFMs in nitrogen atmosphere. (**a**) first DSC scan; (**b**) second DSC scan.

The curing process of the METI NFMs was further investigated by DSC measurements and the results are shown in Figure 7. As shown in Figure 7a, the METI polymers showed a clear two-stage transition process in the first heating scan. In the first stage, the polymers began melting to form endothermic peaks at 230.2 °C, 244.4 °C, and 253.5 °C for METI-5K, METI-10K, and METI-20K samples, respectively. Then, with the increase in temperature, the second stages occurred at 276.1 °C, 301.9 °C, and 323.6 °C for the three METI NFMs, respectively. Apparently, the second transitions were due to the alkynyl crosslinking reactions. It is worth noticing that current METI polymers exhibited crosslinking temperatures (T_c_) in the range of 280–320 °C, which is nearly 70 °C lower than that of the conventional phenylethynyl (C_6_H_4_–C≡C–C_6_H_4_) crosslinker [36]. This would undoubtedly expand the applications of “low-temperature” curable alkynyl-terminated PIs. In addition, the T_c_ values of the METI NFMs increased gradually with an increase in the molecular weights of the polymers, that is, with a decrease in the alkynyl contents of the METI NFMs. Meanwhile, the endothermic peak intensities of the METI polymers also gradually weakened from METI-5K to METI-20K. The second DSC run was recorded and is shown in Figure 7b, in which the exothermic peaks of the thermally crosslinking reactions of METI NFMs all disappeared, indicating complete crosslinking in the polymers. Meanwhile, clear glass transitions were detected. The glass transition temperatures (T_g_) of the three thermally crosslinked METI NFMs were 270.5 °C for METI-5K, 272.0 °C for METI-10K, and 273.5 °C for METI-20K. On one hand, the existence of clear glass transitions in the crosslinked polymers meant that the degrees of the crosslinking reactions for the current METI polymers were relatively low, resulting in some extent of thermoplastic features for the cured METI polymers. On the other hand, the T_g_ values of the three METI polymers were a bit lower than that of the PI-ref (T_g_ = 288.7 °C); however, the molecular weights of the METI polymers had little effect on the molecular segments’ motion at elevated temperatures.

### 3.4. Adhesive Properties

**Figure 8:**
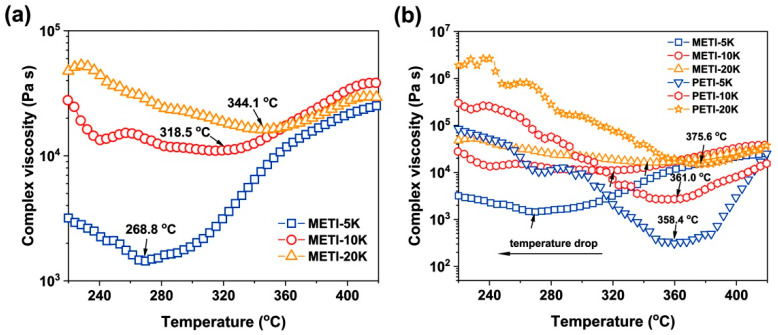
Rheological curves of three molecular weight resins: (**a**) METI; (**b**) METI and PETI.

Generally, the ideal bonding process for the adherends requires the adhesive to have a certain melting flowability to achieve good wettability and infiltrability between the adherends. The determination of complex viscosities and T_c_ of the adhesives play important roles for achieving good adhesion [37]. Thus, rheological behaviors of the METI NFMs were evaluated. As shown in Figure 8a, all METIs exhibited good melting flowability, which was mainly attributed to the controlled molecular weights and also the decreased intermolecular interactions due to the incorporation of flexible ether bonds and bulky phthalide groups into the polymer molecular chains. When METI-5K, METI-10K, and METI-20K samples reached the minimum complex viscosities (η_min_), the corresponding temperatures (T_min_) were 268.8 °C (1413.81 Pa s), 318.5 °C (11,035.2 Pa s), and 344.1 °C (16,187.9 Pa s), respectively. At the same time, the η_min_ values gradually decreased with ta decrease in the molecular weights of the polymers, indicating that the decreased molecular weights were beneficial for the improvement of the melting flowability of the polymers.

The rheological behaviors of the METI NFMs were further compared with the phenylethynyl-terminated (PETI) analogous materials [26] and the results are shown in Figure 8b. On one hand, the T_min_ values of the METIs were apparently lower than those of the PETI counterparts with the same designed molecular weights. For example, the METI-5K exhibited a T_min_ value of 268.8 °C, which was 89.6 °C lower than that of the PETI-5K sample (T_min_ = 358.4 °C). From another point, the temperature required for achieving the same melting viscosity for the METI sample was also significantly lower than that of the PETI sample. The low-temperature crosslinkable feature for the METI NFMs was helpful for the following adhesion applications.

**Figure 9:**
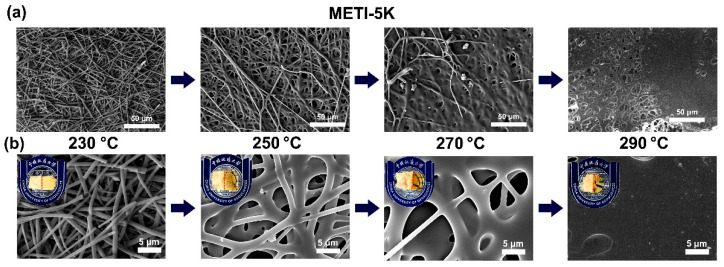
SEM images of METI-5K NFM after hot-pressing treatment. (**a**) 500×; (**b**) 4000×.

**Figure 10:**
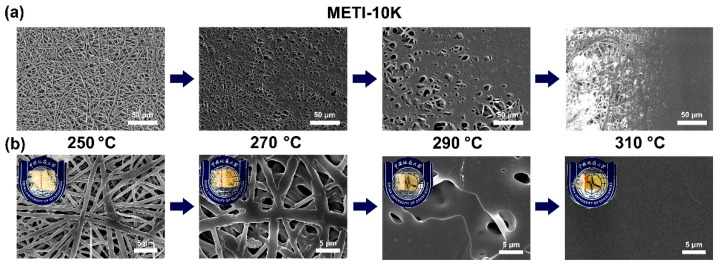
SEM images of METI-10K NFM after hot-pressing treatment. (**a**) 500×; (**b**) 4000×.

**Figure 11:**
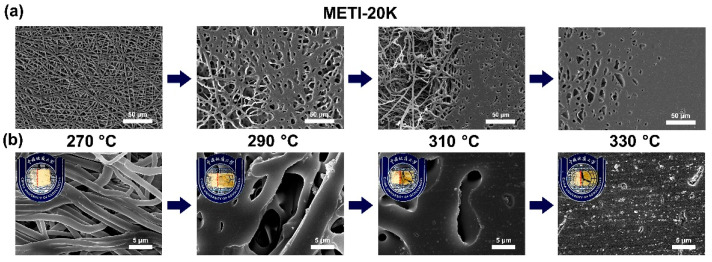
SEM images of METI-20K NFM after hot-pressing treatment. (**a**) 500×; (**b**) 4000×.

To further explore the effects of processing temperature on the melting flowability of the METI NFMs, hot-pressing experiments were performed. The detailed evaluation procedure was reported in our previous work [26]. The double-layer fibrous membranes were placed into a heat-sealing machine, and the pressure was set to 0.5 MPa. The hot-pressing procedure was performed at different temperatures for 60 s. The loading amounts of the METI NFMs on per bonded surface unit were about 30~40 mg. The SEM images of the METI NFMs after hot pressing are shown in Figure 9, Figure 10 and Figure 11. In all three of the figures, the upper left corner of the group (b) shows the appearance of METI NFMs after hot pressing, the left side of the separating red line represents the un-heated part, and the right side is after hot pressing. It can be observed that the fibrous structures of the right part for the NFMs all gradually transformed into uniform film-like structures with increasing hot-pressing temperatures. Meanwhile, the right bi-layer fibrous membranes gradually became transparent, indicting the fusion of the two layers. This interface morphology, which is fibrous on the left and fused film on the right, can be clearly observed in the SEM images shown in Figure 9a, Figure 10a and Figure 11a with a relatively lower magnitude of enlargement.

For the METI-5K sample (Figure 9), when the hot-pressing temperature reached about 290 °C, the two NFM layers were almost completely fused into one uniform film structure; while for the METI-10K and METI-20K samples, the temperatures were 310 °C and 330 °C, respectively. The results were consistent with the rheological test results shown in Figure 8. Compared with our previous work [26], the melting temperatures of METI NFMs were greatly decreased, which was mainly attributed to the lower crosslinking temperature of flexible propynyl groups compared with the rigid phenylethynyl end-cappers. The processing temperatures for the final bonding procedures were then set as follows: METI-5K: 290 °C; METI-10K: 310 °C; and METI-20K: 330 °C.

**Figure 12:**
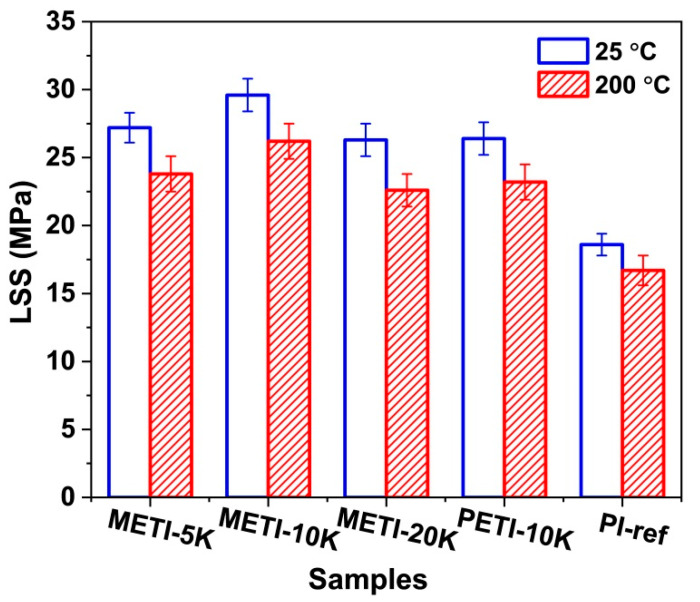
Lap shear strength (LSS) of electrospun METI fiber membranes for bonding stainless steel sheets.

Finally, the single-lap shear strength (LSS) tests were used to evaluate the adhesion performance of the current METI NFMs to the stainless steel adherends [38]. The results are shown in Figure 12, and the data are summarized in Table 4. The three kinds of fibrous membrane adhesives showed good adhesion performance to the stainless steel adherends both at room temperature and at a high temperature of 200 °C. The LSS values were higher than 25 MPa at room temperature and higher than 20 MPa at 200 °C, and were all apparently higher than those of the PI-ref adhesive. This was due to the synergistic effects of the flexible ether linkages, phenolphthalein side chains, and the propynyl terminators. Among them, METI-10K showed the highest bonding strength (LSS_25_ = 29.6 MPa; LSS_200_ = 26.2 MPa) due to its excellent comprehensive properties, including suitable contents of alkynyl end-cappers and moderate molecular weight. Furthermore, the bonding temperature for the METI-10K samples was only 310 °C. Compared with our previous work [26], the processing temperature of the current METI-10K decreases nearly 70 °C lower when compared to the analogous PETI-10K sample (380 °C). The bonding strength was comparable to those of the PETI ones. This would reduce the difficulty in practical bonding fabrications and improve the bonding efficiency.

## 4. Conclusions

Three organo-soluble METI resins with different designed molecular weights were synthesized, based on which, three METI NFM adhesives were prepared via electrospinning technology. The METI NFMs showed good thermal stability, with T_g_ values higher than 270 °C. More importantly, the solvent-free METI NFMs showed “low-temperature” crosslinking features, and could be used as adhesives for stainless steel bonding at relatively low processing temperatures around 290−330 °C. This processing temperature was much lower than the commercially available PETI types of adhesives. In the stainless steel bonding experiments, the METI NFM adhesives all exhibited LSS values higher than 20 MPa. The METI-10K sample showed the best combined properties, including good thermal stability (T_5%_ = 520.2 °C; T_g_ = 276.5 °C), lower melting point viscosity (11035.2 Pa s at 318.5 °C), high bond strength (LSS_25_ = 29.6 MPa; LSS_200_ = 26.2 MPa), and lower crosslinking temperature (T_c_ = 301.9 °C). The information revealed in the current work might be helpful for the research and development (R&D) of high-temperature-resistant adhesives in harsh environments, and could also be beneficial for R&D of low-temperature curable adhesives in aerospace and microelectronic fields.

## Figures and Tables

**Table 1:** Formula for the METI-5K and METI-10K synthesis.

PI	ODPA (g, mol)	BAPPT (g, mol)	MEPA (g, mmol)
METI-5K	10.0015, 32.2410	19.1412, 38.2411	2.2339, 12.0
METI-10K	10.9975, 34.4516	19.2616, 38.4816	1.1170, 6.0

**Table 2:** Inherent viscosities, molecular weights, and solubility of METI and PI-ref resins.

PI	(η)_inh_ ^a^ (dL/g)	Molecular Weight ^b^ (×10^4^ g/mol)			Solubility ^c^				
		M_n_	M_w_	PDI	NMP	DMAc	DMF	CHCl_3_	THF
METI-5K	1.16	1.59	2.17	1.36	++	++	++	+−	+−
METI-10K	1.22	1.71	3.63	2.12	++	++	++	+−	+−
METI-20K	1.01	3.53	5.87	1.66	++	++	++	+−	+−
PI-ref ^d^	1.21	19.0	25.8	1.36	++	++	++	+−	−

^a^ Inherent viscosities measured with PI resins at a concentration of 0.5 g/dL in NMP at 25 °C; ^b^ M_n_: number-average molecular weight; M_w_: weight-average molecular weight; PDI: polydispersity index (M_w_/M_n_); ^c^ ++: Soluble; +−: partially soluble; −: insoluble; CHCl_3_: chloroform; THF: tetrahydrofuran. ^d^ PI-ref: derived from ODPA and BAPPT.

**Table 3:** Thermal properties of METI NFMs.

Samples	T_5%_ ^a^ (°C)	T_10%_ ^a^ (°C)	R_w700_ ^a^ (wt%)	T_g1_^b^ (°C)	T_c_^b^ (°C)	T_g_ ^b^ (°C)
METI-5K	522.5	552.7	64.6	230.2	276.1	274.5
METI-10K	520.2	549.9	64.7	244.4	301.9	276.5
METI-20K	534.8	554.7	65.9	253.5	323.6	276.7
PI-ref	514.1	527.6	59.0	-	-	288.7

^a^ T_5%_: 5% weight-loss temperature; T_10%_: 10% weight-loss temperature; R_w700_: residual weight ratio at 700 °C in nitrogen; ^b^ T_g1_: glass transition temperature without crosslinking in the first scan; T_c_: crosslinking temperature. T_g_: glass transition temperature after crosslinking.

**Table 4:** Bonding strength of METI NFM adhesives.

Samples	LSS_25_ ^a^ (MPa)	LSS_200_ ^a^ (MPa)
METI-5K	27.2	23.8
METI-10K	29.6	26.2
METI-20K	26.3	22.6
PETI-10K ^b^	26.4	23.2
PI-ref	18.6	16.7

^a^ LSS_25_: lap shear strength measures at 25 °C; LSS_200_: lap shear strength measures at 200 °C; ^b^ PETI NFM adhesive with the designed molecular weight of 10,000 g/mol [26].

## Data Availability

The data presented in this study are available on request from the corresponding author.
